# ZIPping to pain relief: the role (or not) of PKMζ in chronic pain

**DOI:** 10.1186/1744-8069-9-6

**Published:** 2013-02-22

**Authors:** Theodore J Price, Sourav Ghosh

**Affiliations:** 1 Department of Pharmacology, The University of Arizona School of Medicine, Arizona, USA; 2 Department of Cellular and Molecular Medicine, The University of Arizona School of Medicine, Arizona, USA; 3 Bio5 Institute, The University of Arizona School of Medicine, Arizona, USA; 4 Graduate Interdisciplinary Program in Neuroscience, The University of Arizona School of Medicine, Arizona, USA

## Abstract

Chronic pain remains a significant clinical problem despite substantial advances in our understanding of how persistent nociceptor stimulation drives plasticity in the CNS. A major theme that has emerged in this area of work is the strong similarity between plasticity involved in learning and memory in CNS regions such as cortex and hippocampus with mechanisms underlying chronic pain development and maintenance in the spinal dorsal horn and other CNS areas such as anterior cingulate cortex (ACC). We, and others have recently implicated an atypical PKC (aPKC), called PKMζ, in the maintenance of pain plasticity based on biochemical assays and the use of a peptide pseudosubstrate inhibitor called ZIP. These studies indicate remarkable parallels between the potential role of PKMζ as a key molecule for the maintenance of long-term memory and long-term potentiation (LTP) and the maintenance of a chronic pain state. On the other hand, very recent studies have disputed the specificity of ZIP and called into question the role of PKMζ as a memory maintenance molecule. Here we critically review the evidence that PKMζ might represent a new target for the reversal of certain chronic pain states. Furthermore, we consider whether ZIP might have other aPKC or even non-aPKC targets and the significance of such off-target effects for evaluating maintenance mechanisms of chronic pain. We conclude that, current controversies aside, utilization of ZIP as a tool to interrogate maintenance mechanisms of chronic pain and further investigations into the potential role of PKMζ, and other aPKCs, in pain plasticity are likely to lead to further insights with the potential to unravel the enigma that is the disease of chronic pain.

## Chronic pain as a disease of CNS plasticity

While plasticity in the CNS is often associated with beneficial processes such as learning and memory, the past decades have brought extensive evidence that disease states, such as addiction and chronic pain also involve CNS plasticity. One of the most commonly studied neurophysiological substrates of this plasticity is long-term potentiation (LTP, [1]). LTP is thought to underlie some forms of learning and memory and, likewise, has been implicated in aspects of addictive behaviors [2] and in nociceptive plasticity [3]. LTP can be divided into an early and late phase with the late phase commencing at least 3 hours after the LTP initiating event. Importantly, these early and late phases of LTP are thought to be governed by different mechanisms [4]. Early-LTP involves the phosphorylation of a variety of ionotropic glutamate receptors mediated by kinases such as classical PKC, PKA and calcium/calmodulin-activated protein kinase II α (CaMKIIα, [5]). For early-LTP to consolidate into late-LTP, these same kinases must be engaged and translation of proteins, mediated largely via the mammalian target of rapamycin (mTOR) pathway, must occur. Once late-LTP has consolidated, inhibition of these same mechanisms is no longer able to reverse established late-LTP [6]. Original theories on the maintenance mechanisms of late-LTP suggested that a persistently active kinase might maintain late-LTP [7]. This idea was eventually supported by evidence that an atypical PKC, PKMζ, that lacks a regulatory region and is therefore, at least after PDK1 phosphorylation, autonomously active, represents the molecular engine for the maintenance of late-LTP and long-term memory [6,8-12].

Some of the first work implicating CNS plasticity in pain amplification came from the description of “central sensitization” by Clifford Woolf in the early 1980s [13]. Since that time, a decade after Bliss and Lomo’s original description of LTP [14], pain neuroscientists have come to recognize the important role that LTP, especially in the spinal dorsal horn, might play in pain plasticity [3,15,16]. Along with this neurophysiological evidence have come a variety of pharmacological studies implicating the same kinases that are involved in early-LTP in spinal pain plasticity. This topic has been extensively reviewed by others, including the myriad similarities and a few important differences [3,15,16]. Similarly, evidence has emerged that pain plasticity leading to chronic pain occurs in other CNS regions that are critical for the processing of nociceptive inputs including, but not limited to, the central nucleus of the amygdala [17,18] and the anterior cingulate cortex (ACC, [19-22]). Hence, it is now clear that the development of a long-lasting pain state involves plasticity in the CNS [23,24]. Moreover, it is also evident that this plasticity, once established, can lead to the transition to a chronic pain state that is resistant to molecular interventions that can be utilized to provide relief of an acute pain state [25-28].

A key question then is how is this chronic pain state maintained and does inhibition of this maintenance mechanism lead to a resolution of the chronic pain state. Herein we will review the evidence that a pseudosubstrate inhibitor of PKMζ, called ZIP, is able to reverse, over differing time courses, a variety of chronic pain states when infused into specific CNS locations [28-33]. These findings yield important insights into how a chronic pain state is maintained and shed light on how the presence of ongoing afferent discharge may differentially regulate plasticity in the CNS. They also suggest that PKMζ may be a key molecular mechanism for pain plasticity in the CNS [31]. However, recent studies have raised serious questions about the specific role of PKMζ in learning and memory and late-LTP maintenance [34,35]. We will argue that careful consideration of these findings opens up a variety of opportunities to gain a better understanding of the mechanism of action of ZIP, the role of aPKC isoforms in CNS plasticity and potential differences between mechanisms governing amplification of pain via CNS plasticity and learning and memory.

## The atypical PKC family, PKCζ, PKCλ and PKMζ

**Figure 1:**
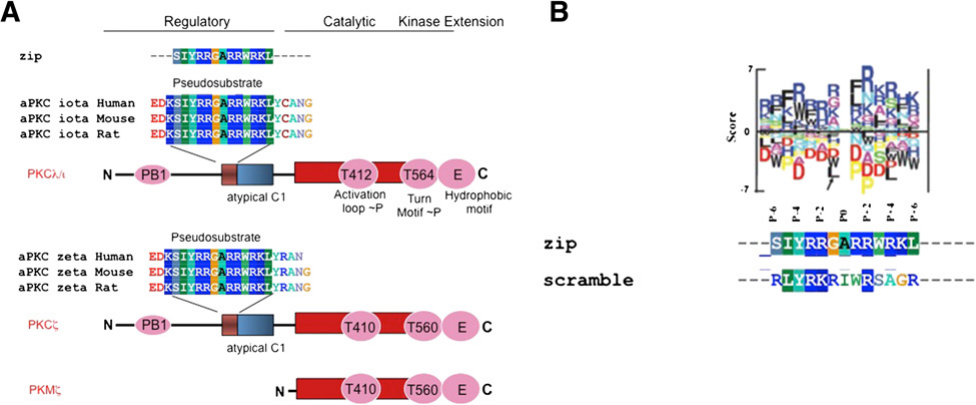
**Schematic of aPKC isoforms and pseudosubstrate inhibitor ZIP: A) The figure shows the protein structure for PKCλ and PKCζ/PKMζ proteins including alignment of pseudosubstrate regions for both genes and conserved phosphorylation sites. **Note that PKMζ is the only aPKC lacking a regulatory region. **B**) Shows the alignment of ZIP and scrambled control peptide seuqneces agaonist the position-specific scoring matrix logo of PKCζ showing some conservation of critical residues [39].

PKC protein kinases are grouped into three major subfamilies: classical PKC, novel PKC and aPKC. There are three major aPKC isozymes in vertebrates: PKCζ, PKCλ (PKCι in human) and PKMζ [36,37]. PKCλ is derived from the *Prkcl* gene while PKCζ and PKMζ are derived from the *Prkcz* gene. PKCζ and PKCλ show a high degree of amino acid sequence identity (~72% amino acid identity overall and ~86% identity between the kinase domain). PKCζ and PKMζ mRNAs originate from the same gene but they have different mRNA structures including an alternative translational start site. All PKCs, except for PKMζ, share the same structural organization – an N-terminal regulatory domain controls the catalytic activation of a C-terminal kinase domain. The mature mRNAs for PKCζ and PKMζ are identical throughout the coding sequence for the catalytic region of the kinase and the 3’ untranslated region but have unique 5’ sequences [38]. PKMζ lacks the regulatory region (summarized in Figure 1A).

Classical PKCs are regulated by intracellular Ca^2+^ and diacylglycerol (DAG) binding at the N-terminal regulatory domain. Novel PKCs are insensitive to intracellular Ca^2+^ but are regulated by DAG. aPKCs, on the other hand, do not respond to either Ca^2+^ or DAG but are regulated by protein-protein interactions and potentially membrane lipid composition. All PKCs, except for PKMζ [38], contain a pseudosubstrate motif in the N-terminal regulatory region – a sequence of amino acids that share identity with PKC substrates, but lacking the phosphoacceptor residue (Figure 1A). This sequence occupies the substrate-binding site in the C-terminal kinase domain and keeps the kinase inactive. Activation of PKCs displaces the pseudosubstrate region and allows substrate binding.

The mechanism of aPKC activation is not entirely clear from a biochemical standpoint. The maturation of newly synthesized PKC requires interaction with HSP90 and a series of priming phosphorylations [40,41]. PDK1 constitutively phosphorylates the “activation loop” of PKCs after synthesis. A second phosphorylation in the “turn motif” of PKC results from autophosphorylation or from phosphorylation by mammalian target of rapamycin complex 2 (mTORC2). A third priming phosphorylation occurs on the “hydrophobic motif”. aPKCs require the activation loop phosphorylation and are regulated downstream of PI3K and PDK1 activity (Figure 1A) [40]. It is interesting to note that classical PKC activation does not require sustained activation loop phosphorylation for activity [42-44]. In fact, this phosphorylation is downregulated in a mature kinase. aPKCs are also phosphorylated in the turn motif [45]. However, the hydrophobic motif in aPKCs contains a glutamic acid instead of the phospho-acceptor residue (Figure 1A). The significance of this residue or the requirement of HSP90 binding for aPKC remains unclear. The unique structure of PKMζ lacking the regulatory domain and the pseudosubstrate sequence is proposed to impart the kinase with constitutive activity or at least sustained activity following PDK1 phosphorylation [6]. Dynamic PKC activation in a cell has been best illuminated by the use of CKARS by Alexandra Newton and colleagues [46-49], and similar analysis of aPKC is likely to shed further light on aPKC activation.

## Role of PKMζ in and effects of ZIP on LTP and memory maintenance

**Figure 2:**
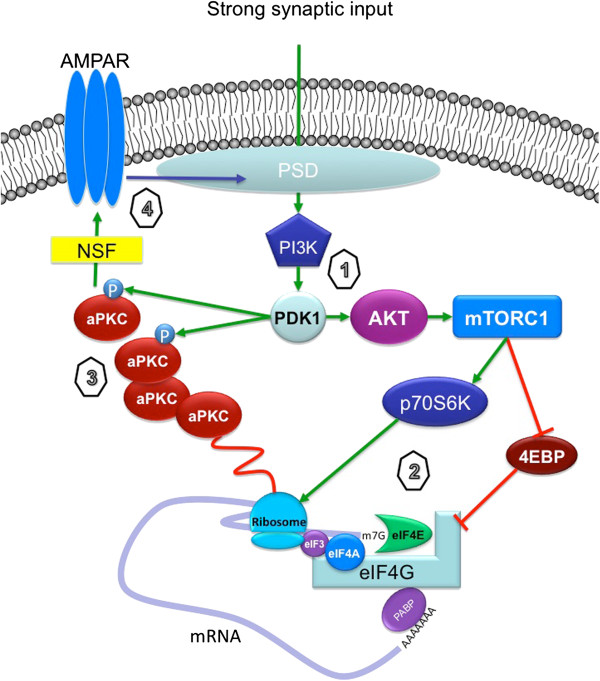
**Proposed role of aPKCs in synaptic plasticity: At most CNS synapses, strong synaptic input leads to an activation of PI3K signaling and PDK1 activation thereby (1) thereby leading to mTORC1 activation. **Engagement of the mTORC1 pathway leads to an increase in translation at or near synaptic sites (2). Because aPKCs mRNAs localize to dendritic and/or synaptic sites, this mTORC1 activation is capable of stimulating nascent synthesis of aPKCs, especially PKMζ (3). Because PDK1 is also activated, this strong synaptic input is linked to increased synthesis and phosphorylation of aPKCs. This enhancement of aPKC number and/or activity at synaptic sites is then linked to increased trafficking of AMPARs to the postsynaptic density (PSD), thereby promoting LTP maintenance.

*A priori* PKMζ is an attractive candidate for LTP maintenance. Its expression is primarily restricted to neurons [50]. It also lacks pseudosubstrate-dependent inhibition [38,51]. This potential autonomous activity suggests that PKMζ is an important player in LTP maintenance. Francis Crick first proposed the idea that a kinase with sustained activity can be the molecule responsible for storage of memory [52] and some evidence was compiled for this idea before a molecular candidate was found [7]. Two decades of work by Todd Sacktor and his colleagues establishes PKMζ as a molecular correlate of LTP maintenance and memory storage [6]. PKMζ was originally thought to be a calpain cleavage-derived kinase active product of the PKCζ protein [53]. This idea, however, was eventually revised when it was recognized that PKMζ could originate from a unique mRNA product expressed in neurons of the brain [38]. This mRNA localizes to dendritic sites [54] and is translated, forming the mature PKMζ, following strong synaptic stimulation [55,56]. This PKMζ synthesis occurs in an mTOR-dependent and ZIP-reversible fashion suggesting a role of PKMζ in regulating its own synthesis [55]. Strong synaptic stimulation is also associated with phosphorylation of PKMζ on two sites and this is regulated by a broad variety of kinases, all of which have been linked to early-LTP and LTP consolidation (Figure 2) [55]. The generation of a pseudosubstrate inhibitor, ZIP (Figure 1B), suggested that PKMζ is required for the maintenance of late-LTP [11]. This was important because previous studies had suggested that a persistently active kinase was required to maintain late-LTP [7,52]. Hence, the structure of PKMζ, combined with inhibitor data gave strong evidence that PKMζ might represent the maintenance mechanism of late-LTP. Subsequent studies demonstrated that ZIP was capable of reversing a wide variety of hippocampal- and/or cortical-dependent learning processes even long after learning was established [57-66]. Eventually, it was shown that overexpression of PKMζ enhanced even established memories whereas expression of a dominant negative PKMζ protein was capable of diminishing such memories [12]. Hence, it was suggested that PKMζ was both necessary and sufficient for the maintenance of late-LTP and learning and memory [6,12,57]. These studies where paralleled by a range of studies indicating that LTP occurs during learning and memory in vivo [67] and that structural changes in dendritic spines accompany both of these processes [68-71].

How, then, does PKMζ maintain late-LTP and long-term memory? It has long been recognized that AMPA receptor trafficking is a critical feature of long-term memory and LTP [5,72]. In line with these observations, PKMζ appears to be involved in trafficking of AMPA receptors, specifically GluA2, to or from the post-synaptic density [57,73,74]. Like most of the electrophysiological and in vivo learning paradigm experiments, some of this evidence was accumulated using the PKMζ inhibitor ZIP. However, peptides that act as inhibitors of n-ethlymaleimide sensitive factor (NSF) –dependent trafficking of AMPA receptors also disrupted AMPA receptor trafficking events associated with PKMζ-dependent learning events [74]. Moreover, the effects of ZIP on removal of GluA2 from the synapse could be occluded by peptides that interfered with GluA2 removal from synaptic sites [73]. Hence, the consensus emerged that PKMζ shifts the balance of AMPA receptor trafficking away from removal from the synapse toward AMPA receptor movement toward the synapse (Figure 2) [6]. Consequently, inhibition of PKMζ with ZIP leads to removal of GluA2 from synaptic sites [73]. The idea has since emerged that LTP involves insertion of glutamate (AMPA or kainate) receptors into the synaptic membrane independently of receptor subtype and the GluA1 C-terminus, which was thought to be crucial for LTP. This finding suggests that LTP simply requires a reserve pool of glutamate receptors that are available to be shifted toward the post-synaptic density [75]. It remains to be seen if ZIP disrupts the trafficking or removal of a wide range of ionotropic glutamate receptors form the synapse.

Hence, the data discussed above points to a model wherein synaptic stimulation sufficient to lead to LTP causes an mTOR-dependent synthesis of PKMζ and phosphorylation of the protein mediated by PDK1 (the kinase for the T410 site). This synthesis of PKMζ is associated with an increase in NSF-dependent trafficking of GluA2 to the synapse and the use of ZIP disrupts the retention of GluA2 in the synapse [6,57]. Therefore, this model, which must now be questioned based on data presented below [34,35], suggests that PKMζ initiates and maintains late-LTP and learning and memory by shifting the balance of AMPA receptors toward accumulation at the synapse.

## Role of PKMζ in and effects of ZIP on pain plasticity

As mentioned above, CNS plasticity is well recognized as a mechanism of pain amplification and this plasticity is thought to underlie the development of many chronic pain states [3,15,23,24,76]. However, it has only recently been suggested that PKMζ might play a role in this plasticity. The first evidence for this came from the labs of Min Zhuo and Bong-Kiun Kaang in 2010 [29]. Using a nerve injury model in mice, these authors showed that peripheral nerve injury is accompanied by an early increase in PKMζ expression and a persistent increase in PKMζ phosphorylation in the ACC. Consistent with a role for PKMζ in CNS plasticity leading to neuropathic pain, ZIP infusion into the ACC relieved mechanical allodynia in these mice and led to a conditioned place preference suggesting relief of spontaneous pain via an ACC, PKMζ-related mechanism. Finally, these authors demonstrated that ZIP exposure to ACC slices prepared from neuropathic animals led to a decrease in AMPA receptor-mediated currents, whereas ZIP had no effect in sham mice. This finding is consistent with the notion that peripheral nerve injury leads to the insertion of AMPA receptors in a ZIP-reversible fashion, similarly to observations in other cortical or hippocampal areas in learning paradigms [73,74,77]. Somewhat surprisingly, in this work, there was no effect of ZIP when it was infused into the spinal cord in neuropathic animals and ZIP failed to distinguish between AMPA receptor current densities between neuropathic and sham animals, although it suppressed these currents in both groups. Hence, at least in the case of neuropathic pain, a ZIP-reversible form of plasticity in the ACC appears to be a key feature of this pain state whereas the spinal cord plays only a minor role [29]. Subsequent studies from Sandkuhler’s group suggested that ZIP does not reverse late-LTP at C-fiber synapses in the outer lamina of the dorsal horn [78]. Hence, there may be fundamental differences between the effects of ZIP in hippocampus and cortex vs. this synapse in the dorsal horn. This possibility and its implications will be discussed below.

These findings in the ACC are further supported by data from our labs, in collaboration with Frank Porreca. We found that, in rats, PKMζ phosphorylation is increased in the rostral ACC (rACC) and rACC infusion of ZIP leads to a long lasting (at least 3 days, and up to 7 days long) reversal of ongoing neuropathic pain [33]. In contrast to findings in mice, we did not observe any change in neuropathic allodynia over this same time course when ZIP was infused into the rACC of rats. This discrepancy is difficult to rectify but may be due to a species difference between the neuroanatomical segregation of tonic aversive and sensory discriminative aspects of pain insofar as ACC treatments and/or lesions have reliably shown no effect on tactile thresholds in rats whereas they demonstrate robust relief of tonic aversive aspects of pain across multiple models in this species. On the other hand, several groups have demonstrated relief of tactile hypersensitivity in mice with ACC treatments, especially in neuropathic models. In the spinal cord, we also failed to observe any change in PKMζ protein levels or phosphorylation after peripheral nerve injury. Moreover, spinal infusion of ZIP failed to influence mechanical allodynia or spontaneous pain evoked by spinal nerve ligation (SNL) surgery. On the other hand, ZIP treatment did lead to a transient reversal of thermal hyperalgesia [33]. Since the presence of neuropathic allodynia after nerve injury has been shown to persist even after the ablation of all nociceptive fibers in mice, this finding, which has now been replicated in the chronic constriction injury (CCI) model [30], suggests that this form of allodynia is not dependent on a ZIP-reversible process in the spinal cord. Thermal hyperalgesia, on the other hand, appears to be dependent on a spinally-encoded, ZIP-reversible process [33]. Hence, a ZIP-reversible form of plasticity contributes to key features of neuropathic pain and this is positively correlated with a long-lasting increase in phosphorylation of PKMζ, but not increased synthesis, in the ACC of mice and rats.

In contrast to neuropathic pain, a spinal, ZIP-dependent process appears to be crucial to other types of chronic pain and this plasticity is, in some cases, paralleled by changes in PKMζ phosphorylation and synthesis. We sought out to understand whether PKMζ might be involved in maintaining a chronic pain state utilizing models of hyperalgesic priming pioneered by Jon Levine and colleagues [25-27,79-81]. Hyperalgesic priming models involve the exposure to an algogen or an inflammatory mediator followed by a brief period of hyperalgesia or allodynia. The “primed” animal is then exposed to a low dose of an inflammatory mediator, such as prostaglandin E_2_ (PGE_2_) which fails to promote a state of tactile hypersensitivity in naïve animals but in the primed animal elicits a long-lasting (at least 24 hrs) state of hypersensitivity. This model, therefore, has the advantage of a clearly delineated initiation phase (priming) followed by a period of maintenance with no outward signs of hypersensitivity until a low dose inflammatory mediator is given to elicit a state of hypersensitivity. Building on existing data showing that interleukin-6 (IL-6) can induce such priming in rats [80], we demonstrated that this effect can be reproduced in mice [28,82]. Matching initial injections of IL-6 into the paw with intrathecal injection of specific kinase inhibitors demonstrated that initiation mechanisms in this model are very consistent with similar studies conducted in hippocampal learning tasks. Hence, initiation of priming is mTOR-, CaMKIIα- and classical PKC-dependent. However, a much different picture emerges when these same inhibitors are utilized during the maintenance phase of hyperalgesic priming when these same doses fail to reverse the exaggerated response to inflammatory mediator exposure. On the other hand, ZIP treatment, either during the initiation or maintenance phase completely reverses the effects of priming on subsequent exposure to the inflammatory mediator [28]. Consistent with a role for AMPA receptor trafficking in the persistence of this priming effect, a peptide that disrupts NSF-dependent AMPA receptor trafficking [74] mimics the effects of ZIP. This, then, is consistent with a PKMζ-dependent maintenance mechanism for hyperalgesic priming. Importantly, this is not a peculiarity of the IL-6 priming model as an identical pharmacological pattern is produced with plantar incision as the priming stimulus. The effects are also independent of the precipitating stimulus as centrally-mediated, mGLuR1/5-dependent precipitation of exaggerated nocifensive responses are also ZIP- and AMPA receptor trafficking-dependent in the maintenance phase [28]. Hence, although hyperalgesic priming has a strong nociceptor plasticity-dependent component, the spinal cord encodes an engram for precipitation of a long-lasting hypersensitivity following priming that is ZIP-reversible, suggesting a potential role for PKMζ. This notion is further supported by the finding that virally-mediated expression of a membrane targeted PKCζ, making the protein constitutively active, like PKMζ, recapitulates hyperalgesic priming behavior without the priming event. Therefore, similarly to overexpression of PKMζ enhancing learning and memory, overexpression of a PKMζ mimetic is sufficient to achieve a long-lasting state of spinally-mediated pain plasticity [28].

A potential role for PKMζ in spinal pain amplification is not limited to the hyperalgesic priming model. Two groups have now demonstrated that ZIP injection into the spinal cord leads to an inhibition of the 2^nd^ phase formalin-induced nocifensive behaviors [30,32], even when ZIP is administered following the cessation of the 1^st^ phase [30]. This is also positively correlated with an increase in PKMζ phosphorylation [32] and an increase in total PKMζ levels in the spinal dorsal horn [30]. Paralleling these findings in the formalin test, intraplantar capsaicin treatment specifically stimulates an increase in dorsal horn PKMζ levels among aPKCs and ZIP leads to a reversal of capsaicin-evoked mechanical allodynia [30]. Importantly, these effects are not limited to behavioral manifestations as ZIP, but not scrambled ZIP, administration to the spinal cord inhibits formalin-induced action potential firing of wide dynamic range (WDR) neurons [32] and capsaicin-evoked mechanical hypersensitivity of WDR neurons [30]. Finally, ZIP administration to the spinal cord reverses wholesale inflammation-induced thermal and mechanical hyperalgesia, albeit transiently, and likewise decreases spinal c-FOS expression [32]. Importantly, using a model of chronic post ischemic pain (CPIP) that is initially dependent on afferent discharge but transitions to a centrally-maintained chronic pain state, Laferriere and colleagues demonstrate that spinal administration of ZIP during the centrally-maintained phase of the model leads to a complete and seemingly permanent reversal of mechanical hypersensitivity [30]. This observation is highly compatible with findings in the hyperalgesic priming model [28] and suggests that a centralized chronic pain state, that is no longer dependent on afferent discharge, is reversible by a single infusion of ZIP into the spinal cord [28,30].

## Regulation of PKMζ phosphorylation and synthesis

The regulation of aPKC phosphorylation has been the topic of intensive investigation and it is now well understood that T410 phosphorylation is mediated by PDK1 [83] whereas T560 is an autophosphorylation and/or mTORC2 site [84] and these sites also appear to be the key residues for regulation of PKMζ. Synthesis of PKMζ is regulated by a variety of factors, however, the key step in this process, for activity-dependent translation, is stimulation of the mTOR pathway [55], consistent with a key role for mTOR in the initiation of synaptic plasticity [85] (including pain plasticity [28,86]). While these intracellular mediators are known, extracellular factors involved in the regulation of PKMζ have been harder to pin down.

Over the past two years one key factor has emerged, brain derived neurotrophic factor (BDNF). BDNF is an important molecule for synaptic plasticity in the CNS [87-89] and has been linked to diverse processes including learning and memory [87-89], addiction [90-92] and pain plasticity [93-95]. BDNF and PKMζ, based on data obtained with ZIP, cooperate to govern metaplasticity in the hippocampus [96]. Moreover, BDNF signaling via its receptor, trkB, engages a ZIP reversible phosphorylation of a palmitoylation enzyme called ZDHHC8 involved in the synaptic localization of post synaptic density protein 95 (PSD95) [97]. In the spinal cord, we have recently shown that exposure of spinal synaptosomes to BDNF leads to an increase in nascent synthesis of PKMζ and PKCλ that is dependent on mTOR activation [98]. BDNF similarly increases PKMζ phosphorylation at T410. Spinally applied BDNF promotes hyperalgesic priming that is reversible by spinal infusion of ZIP suggesting a link to a functional role of PKMζ and/or PKCλ in BDNF-induced chronic pain [98]. Interestingly, our findings with BDNF inhibitors, applied either spinally or systemically, indicate that BDNF signaling through trkB plays an essential role in the maintenance of hyperalgesic priming. Hence, these findings collectively indicate a key role for BDNF in not only the initiation of chronic pain states (as has been shown for inflammatory pain [99]) but also in the maintenance of such pain states [98]. They also implicate an active role for BDNF in regulating PKMζ and/or PKCλ during the maintenance phase of hyperalgesic priming suggesting that therapeutic strategies wherein a single treatment with BDNF signaling disrupting agents might be capable of permanently reversing a centralized chronic pain state.

Another tyrosine kinase receptor-linked pathway may play an important role in regulating PKMζ, activation of nerve growth factor (NGF) signaling via trkA or p75 [100]. In contrast to the studies mentioned above, this pathway has been implicated in the regulation of excitability of peripheral nervous system (PNS) neurons of the dorsal root ganglion (DRG). Here it has long been understood that NGF alters the excitability of adult DRG neurons but downstream mechanisms involved in this effect are still under investigation. Zhang et al., demonstrated the NGF stimulated an enhanced excitability of DRG neurons that was reversible by ZIP and PI3K inhibitors. Interestingly, this enhanced excitability was also blocked by siRNA treatments that decreased PKMζ but not PKCζ or PKCλ expression, suggesting a specific role for PKMζ in this effect [100]. Moreover, infusion of recombinant PKMζ recapitulated the effect of NGF. Hence, NGF appears to regulate DRG excitability via a PKMζ-dependent process.

Another receptor system crucial for regulation of PKMζ is the group I metabotropic glutamate receptor family (mGluR1/5). First, DHPG, an agonist of these receptors, permits for metaplasticity in a ZIP-dependent fashion suggesting a role for mGluR1/5 in regulation of PKMζ [96]. More direct evidence comes from work done examining the effects of DHPG in the spinal cord. Activation of spinal mGluR1/5 receptors stimulates nocifensive behavior and long-lasting mechanical hypersensitivity that has hitherto been largely attributed to MAPK, specifically ERK, activation [28,86,101-105]. However, spinal activation of mGluR1/5 receptors with DHPG also stimulates a long lasting increase in total PKMζ levels [30]. Moreover, DHPG-induced allodynia is completely reversed by spinal administration of ZIP [30] suggesting that mGluR1/5-mediated mechanical hypersensitivity is maintained by a persistent increase in PKMζ levels. Hence, in the pain pathway, as well as in crucial learning and memory circuits, BDNF/trkB [98] and mGluR1/5 [28,30] appear to act as key regulators of PKMζ synthesis, phosphorylation and their downstream physiological consequences.

## ZIP as a specific inhibitor of PKMζ

As described above, investigators examining the potential role of PKMζ in synaptic plasticity and accompanying behavioral manifestations of such plasticity have relied heavily on ZIP as a tool to interrogate the function of PKMζ (Figure 1B). Hence, this area is highly dependent on the specificity of ZIP as a tool to inhibit PKMζ. This specificity has recently been called into question on several fronts [106,107]. First, an investigation of PKMζ expressed in a heterologous systems or examining native activity in brain slices found that ZIP failed to block kinase activity of the enzyme [106], however, a subsequent report disputed some of the conclusions posited by Wu-Zhang and colleagues [108]. Furthermore, a scrambled peptide is routinely used as negative control. However, assessment of binding interface determined by positional scanning of oriented peptide libraries indicates that the control peptide may bind to aPKCs, including PKMζ (Figure 1B). Indeed, recent reports [34,35] and our unpublished observations confirm the lack of specificity and isoform selectivity *in vitro*. The basis of its lack of PKMζ inhibition *in vivo* remains unknown. Chelerythrine, a benzophenanthridine alkaloid which is reported to inhibit PKMζ inhibition *in vivo*[53] fails to inhibit aPKC *in vitro*[106,109]. While the controversy over the activity of ZIP against kinase activity in cells (versus in vitro assays) continues [106,108], two recent papers have raised the specter that ZIP possesses targets other than PKMζ and that these targets may, in fact, represent the mechanism of action of ZIP for disruption of late-LTP and long-term memory [34,35]. The derivation of ZIP sequence from the autoinhibitory pseudosubstrate peptide sequence of PKCζ ostensibly confers its specificity for PKCζ and PKMζ. The pseudosubstrate sequence of PKCλ is identical to ZIP (Figure 1A). The PKC substrate peptide often used in *in vitro* kinase assay also shares substantial identity. Finally, ZIP inhibits PKCλ in *in vitro* kinase assays [35]. The most definitive proof of “off-target” effect however is the ability of ZIP to affect memory storage in PKMζ knockout mouse [34,35]. Notwithstanding, these studies suggest that ZIP functions in erasing memory storage by perturbing the biological activity of an alternative target.

## Functional redundancy and speculations on alternative targets of ZIP

While genetic ablation of PKMζ clearly establishes that it is not necessary for LTP maintenance and memory storage [34,35], these studies do not rule out PKMζ function in memory. Fundamental processes in biology, such as maintenance mechanisms of LTP, most likely involve functional redundancy of signaling pathways and components. A key cellular function of aPKCs is in the regulation of cell polarity. Here, as part of the Par complex, aPKCs establishes asymmetry within a cell, including during the first cell division in *C*. *elegans* embryo. Functional analogy between polarity and memory roles of aPKCs have been indicated [57]. A recent RNAi screen in *C*. *elegans* demonstrates that functional redundancy of the signaling network masks the function of individual polarity components [110]. Single gene knockout studies can often overlook important function of molecules in complex physiological processes. One particularly striking example is the Tyro3, Axl and Mer family of receptor tyrosine kinases. The genetic ablation of all three members was necessary to fully reveal their biological function [111]. A similar redundancy between PKC isoforms is conceivable.

Another possibility is that the ZIP-mediated effect on LTP maintenance in wild-type and PKM/PKCζ knockout mice occurs not because of its ability to target PKMζ, but its efficacy in targeting an unidentified protein. This elusive protein is perhaps necessary and sufficient for LTP maintenance. So what may be the molecule that accounts for redundancy or is the elusive, true memory storage molecule? The aPKC isoform – PKCλ, is expressed in neurons, including in the hippocampus, cortex, and amygdala ( [112] and our unpublished data) and is inhibited by ZIP with the same kinetics as PKMζ ( [35] and our unpublished data). It has also been reported that overexpression of PKMζ enhances, while expression of kinase-inactive PKMζ functions as dominant negative in LTP maintenance [12]. Because the PKCλ kinase domain shares 86% identity at the amino acid level with PKMζ, it is likely that many of the molecular targets will be shared between these isoforms, particularly during overexpression. Therefore, it is not outside the realm of possibility that genetic deletion of PKM/PKCζ, as has recently been done [34,35], reveals a functionally redundant and crucial role of PKCλ in maintenance of late-LTP and long term memory storage. Conditional knockout of PKCλ (interestingly, conventional knockout of PKCλ is lethal [113,114], while many other PKCs are dispensable for life) or isoform-selective inhibitors merit testing for effects on memory storage and chronic pain. A systematic analysis of ZIP targets, based on predicted homology or unbiased screens, combined with genetic knockouts may yet reveal the secret of the elusive memory molecule.

## Deciphering the effects of ZIP and the role of PKMζ in pain plasticity

There are several possible ways to interpret the studies mentioned above demonstrating a lack of specificity of ZIP for PKMζ in late-LTP maintenance and long-term memory storage in relation to their relevance for studies examining pain plasticity. Below we will consider some of those possible interpretations and their ramifications for understanding the role of aPKCs in pain plasticity.

**Figure 3:**
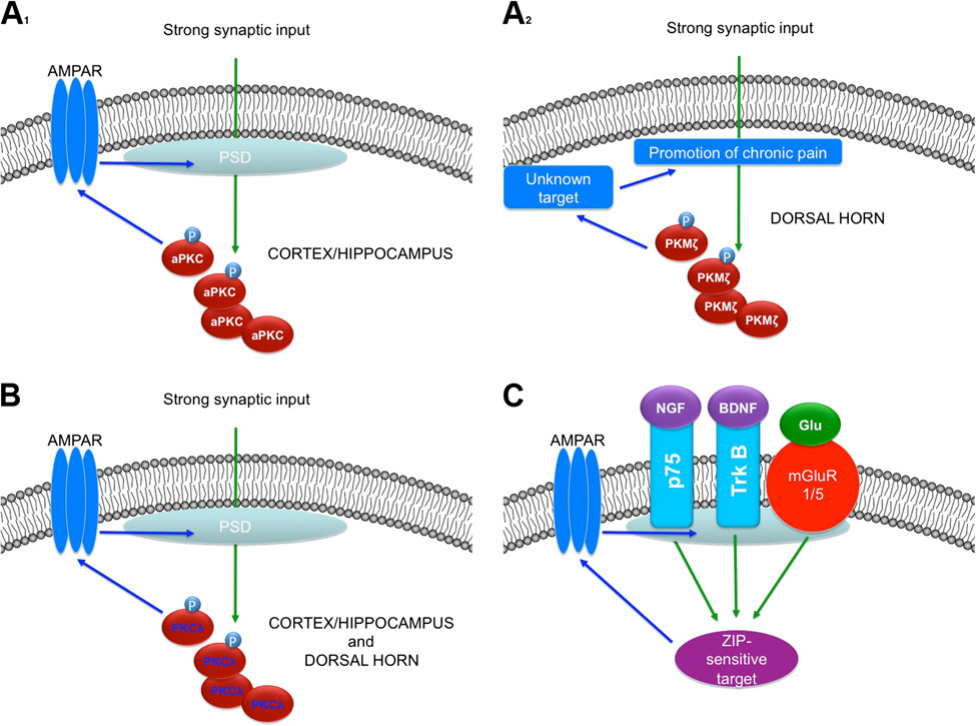
**Three hypotheses regarding the role of aPKCs in pain plasticity taking into account recent findings on PKMζ knockout mice: A_**1 **_and A_**2**_) One possibility is that while cortical and hippocampal late-LTP is dependent on aPKC function through trafficking of AMPA receptors (AMPAR) this mechanism is not shared in the dorsal horn. **In the dorsal horn PKMζ might be necessary and sufficient for certain forms of pain plasticity but this is not functionally linked to LTP or AMPAR trafficking and therefore involves a novel, undiscovered mechanism. Another possibility (**B**) is that at all CNS synapses aPKCs can serve a functionally redundant role and the absence of PKMζ fails to lead to a strong phenotype because PKCλ, which is also ZIP-sensitive, can functionally replace PKMζ in its absence. **C**) A final possibility is that ZIP targets a totally distinct mechanism to induce neuronal plasticity, however, such a mechanism is likely stimulated by NGF/p75-, BDNF/trkB- and mGluR1/5-dependent mechanisms that may also, in some cases, be linked to AMPAR trafficking as an endpoint signaling output of the ZIP-sensitive target.

1) It is possible that PKMζ is the sole target for ZIP in the pain pathway and that studies examining hippocampal and cortical effects of ZIP will ultimately not be paralleled by spinal ZIP application studies (Figure 3A_1_ and 3A_2_). In many ways this result would be very exciting for the development of therapeutics because it would suggest that small molecule inhibitors of PKMζ could be developed for inhibition or reversal of pathological pain plasticity that would not have an influence, necessarily, on learning and memory. While this possibility may sound improbable based on the literature discussed above, there are some important points to consider. First, as mentioned above, there is already some evidence that ZIP fails to reverse late-LTP at synapses between C-fibers and second order outer lamina neurons [78]. This occurs despite the fact that ZIP has clear and robust effects in several pain models [28-30,32,33,98], including a complete reversal of a centralized pain state with a single dose in hyperalgesic priming and CPIP models [28,30,98]. Hence, ZIP may have an effect on physiological process that are distinct from LTP in the spinal cord but that are nevertheless crucial for pain plasticity. In that regard, it is important to note that ZIP reverses hyperalgesic priming even when priming-induced allodynia has completely resolved [28,98]. While the pharmacology of this event is consistent with the pharmacology of early- vs. late-LTP, the mere fact that the allodynia resolves questions the relevance of LTP in this model, especially at afferent / second order neuron synapses.

2) Another possibility is that functional redundancy of aPKCs is a key feature of pain plasticity in a similar fashion to what might well be observed in learning and memory processes (Figure 3B). This is, from an evolutionary perspective, a tantalizing possibility especially considering the important teaching function that the nociceptive system possesses for the survival of complex organisms [115]. As mentioned above, the fact that ZIP has inhibitory activity at PKCλ, combined with the demonstrated activity of ZIP in PKM/PKCζ knockout mice [34,35], point to the clear need for experiments aimed at assessing a potential role for PKCλ as an important molecule for synaptic plasticity in multiple systems and pathways. In this regard, it should not be forgotten that overexpression of aPKCs in memory [12] or pain circuits [28] is sufficient to enhance memory or induce a chronic pain state, respectively. Moreover, we have shown that PKMζ and PKCλ are regulated in a similar fashion at spinal synapses [98]. We are unaware of other scenarios where a class of enzyme is, on the one hand, sufficient for an effect whereas it is, on the other hand, not necessary for the same effect. Based on these factors, we favor the functional redundancy hypothesis as the most parsimonious, albeit untested, solution to this problem.

3) The final possibility is that ZIP has a mechanism of action that is completely unrelated to aPKC function (Figure 3C). If this is true, it is interesting to consider that a wide variety of other kinases have already been ruled out due to extensive investigations of maintenance mechanisms of late-LTP, memory storage and chronic pain. It is highly likely that such a mechanism would need to also involve the trafficking of AMPA receptors (or at least a reserve pool of ionotropic glutamate receptors [75]) because several previous studies have linked ZIP and PKMζ effects to this process [28,73,74]. It is also probable that such a mechanism should also be regulated by mGluR1/5 [30] and BDNF/trkB signaling [98] (and likely NGF signaling as well [100]) since the physiology of these pathways has been linked to ZIP reversible processes. Having said that, it is still likely that the most judicious path forward in this regard is the undertaking of true unbiased screens to determine potential molecular partners of ZIP action outside of the aPKC family of kinases.

## Concluding remarks

The recent introduction of PKMζ into the pantheon of pain targets has led to new insights into how pain becomes chronic while also unveiling new mysteries of pain physiology (e.g. clear differences in plasticity mechanisms between ongoing neuropathic and centralized chronic pain states). New studies demonstrating a lack of specificity of the central tool in these experiments, ZIP, have, in some ways, turned this area on its head; however, from another perspective, this may be exactly what this area of work needs. We propose that this field is now ripe for discovery and the development of hitherto unimagined tools that will dramatically enhance our understanding of the role, or lack thereof, for aPKCs in fundamental neurobiological processes like pain plasticity. We look forward to exciting discoveries in this now completely wide-open area of work in the coming years.

## Competing interests

The authors declare that they have no competing interests.

## Authors’ contribution

TJP and SG wrote the mauscript. All authors read and approved the final manuscript.
